# Vitamin D and Rheumatic Diseases: A Review of Clinical Evidence

**DOI:** 10.3390/ijms221910659

**Published:** 2021-10-01

**Authors:** Nipith Charoenngam

**Affiliations:** 1 Department of Medicine, Mount Auburn Hospital, Harvard Medical School, Cambridge, MA 02138, USA; nipith.charoenngam@gmail.com; 2 Section Endocrinology, Diabetes, Nutrition and Weight Management, Department of Medicine, Boston University School of Medicine, Boston, MA 02118, USA; 3 Department of Medicine, Faculty of Medicine Siriraj Hospital, Mahidol University, Bangkok 10700, Thailand

**Keywords:** vitamin D, 25-hydroxyvitamin D, 1,25-dihydroxyvitamin D, rheumatic diseases, rheumatology, rheumatoid arthritis, systemic lupus erythematosus, spondyloarthropathies, osteoarthritis, hyperuricemia, gout

## Abstract

Vitamin D plays an important role in maintaining a healthy mineralized skeleton. It is also considered an immunomodulatory agent that regulates innate and adaptive immune systems. The aim of this narrative review is to provide general concepts of vitamin D for the skeletal and immune health, and to summarize the mechanistic, epidemiological, and clinical evidence on the relationship between vitamin D and rheumatic diseases. Multiple observational studies have demonstrated the association between a low level of serum 25-hydroxyvitamin D [25(OH)D] and the presence and severity of several rheumatic diseases, such as rheumatoid arthritis (RA), systemic lupus erythematosus (SLE), spondyloarthropathies, and osteoarthritis (OA). Nevertheless, the specific benefits of vitamin D supplements for the treatment and prevention of rheumatic diseases are less accepted as the results from randomized clinical trials are inconsistent, although some conceivable benefits of vitamin D for the improvement of disease activity of RA, SLE, and OA have been demonstrated in meta-analyses. It is also possible that some individuals might benefit from vitamin D differently than others, as inter-individual difference in responsiveness to vitamin D supplementation has been observed in genomic studies. Although the optimal level of serum 25(OH)D is still debatable, it is advisable it is advisable that patients with rheumatic diseases should maintain a serum 25(OH)D level of at least 30 ng/mL (75 nmol/L) to prevent osteomalacia, secondary osteoporosis, and fracture, and possibly 40–60 ng/mL (100–150 nmol/L) to achieve maximal benefit from vitamin D for immune health and overall health.

## 1. Introduction

Vitamin D is a steroid hormone responsible for the regulation of calcium and phosphate metabolism and for maintaining a healthy mineralized skeleton [1,2,3]. In addition, it is known to exert various non-skeletal actions due to the presence of the vitamin D receptor (VDR) in most tissues, including the skin, adipose tissue, skeletal muscle, endocrine pancreas, immune cells, breast, blood vessels, and brain [1,2,4].

Rheumatic diseases are a spectrum of autoimmune and/or inflammatory diseases that cause damage to joints, muscles, and bones, as well as vital organs such as the lungs, heart, kidneys, and nervous system. In rheumatology, vitamin D supplementation is recommended to prevent glucocorticoid-induced osteoporosis and to reduce the risk of fracture in patients with osteoporosis [5]. It is proposed that the improvement of vitamin D status may help protect against the development and severity of rheumatic diseases, given the specific actions of vitamin D on the skeletal and immune systems [2,6,7]. The purpose of this review is to provide general concepts of vitamin D for the skeletal and immune health, and to summarize the mechanistic, epidemiological, and clinical evidence on the relationship between vitamin D and several types of rheumatic diseases, including rheumatoid arthritis (RA), systemic lupus erythematosus (SLE), spondyloarthropathies (SpA), gout and hyperuricemia, osteoarthritis (OA), and others.

## 2. Physiology of Vitamin D

**Figure 1:**
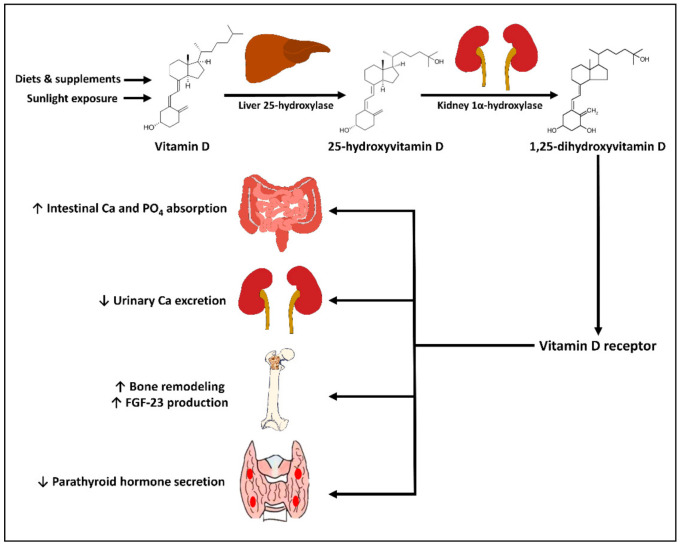
Schematic representation of the synthesis, sources, and metabolism of vitamin D for skeletal function. ↑: Increased; ↓: Decreased; Ca: Calcium; FGF-23: fibroblast growth factor-23; PO_4_: phosphate.

Humans gets vitamin D from dietary consumption, supplements, and endogenous synthesis in the skin. The two major forms of vitamin D are vitamin D_2_ and vitamin D_3_. Vitamin D_2_, synthesized from ergosterol, can be found in ultraviolet irradiated and sundried mushrooms and yeasts. As shown in Figure 1, vitamin D_3_, synthesized from 7-dehydrocholesterol, can be found in animal products such as cod liver oil and oily fish, and is synthesized endogenously in the skin [1,2,3]. After entering circulation, vitamin D (D_2_ and D_3_) is metabolized in the liver by the enzyme vitamin D-25-hydroxylase (CYP2R1) to 25-hydroxyvitamin D [25(OH)D], which is the major circulating form of vitamin D that is clinically measured to reflect vitamin D status [1,2,8]. Circulating 25(OH)D is then further metabolized by the enzyme 25-hydroxyvitamin D-1α-hydroxylase (CYP27B1) to 1,25-dihydroxyvitamin D [1,25(OH)_2_D], the biologically active form. 1,25(OH)_2_D exerts its functions in the target tissue by binding to the vitamin D receptor (VDR) in the nucleus, where it triggers the up- or down-regulation of multitudes of genes in multiple types of tissues including renal tubular cells, intestinal epithelium, parathyroid glands, bone cells, and immune cells [1,2,3,8]. Those include genes involved in calcium and phosphate metabolism, and genes associated with risks of certain autoimmune diseases [1,9,10].

The main site of conversion of 25(OH)D into the systemically bioavailable 1,25(OH)2D is the kidneys, where CYP27B1 is expressed and regulated by parathyroid hormone (PTH) and fibroblast growth factor-23 (FGF-23) [11]. CYP27B1 expressed by many other tissues (e.g., immune cells, parathyroid glands, microglia, breast, colon, and keratinocytes) can also convert 25(OH)D into 1,25(OH)_2_D, resulting in intracrine and paracrine signaling, without being regulated by PTH or FGF-23 [12]. Both 25(OH)D and 1,25(OH)_2_D are metabolized by the enzyme 24-hydroxylase (CYP24A1), expressed mainly by the intestine, bone, and kidneys into inactive water-soluble carboxylic acids, which are then excreted in the bile [13].

## 3. General Concepts of Vitamin D for Skeletal and Immune Health

### 3.1. Effects of Vitamin D on Bone and Mineral Metabolism

Vitamin D exerts its effects on bone and mineral metabolism mainly by altering the expressions of several genes in the small intestine, kidneys, parathyroid glands, and bone [2,3]. Activation of VDR by 1,25(OH)_2_D promotes intestinal calcium and phosphate absorption and renal tubular calcium reabsorption, which help maintain an adequate calcium−phosphate product that crystallizes in the collagen matrix in the bone. 1,25(OH)_2_D also has direct effects on the bone by stimulating the receptor activator of nuclear factor kappa-B-dependent bone resorption and inducing the expression of osteocalcin, the major non-collagenous protein in the skeleton [14,15,16]. Furthermore, 1,25(OH)_2_D directly inhibits PTH production, leading to decreased bone resorption, and induces FGF-23 production by the osteocytes, leading to increased urinary phosphate excretion [11,17,18].

**Figure 2:**
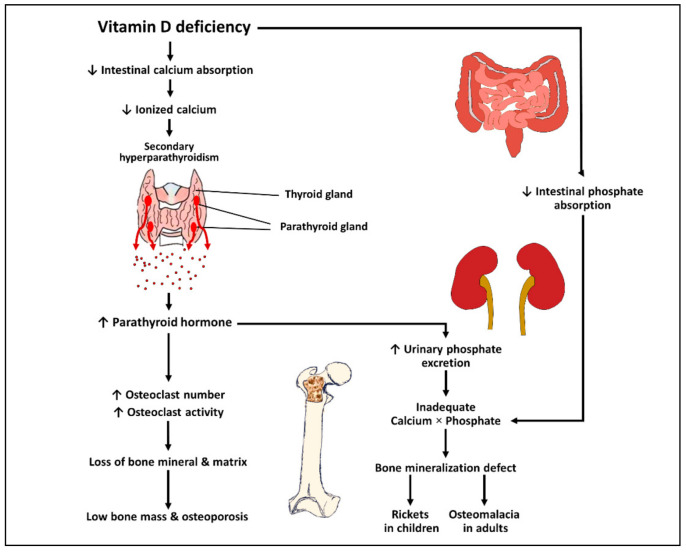
Schematic representation of the pathophysiology of vitamin D deficiency causing rickets, osteomalacia, and osteoporosis. ↑: Increased; ↓: Decreased.

The pathophysiology of vitamin D deficiency causing rickets, osteomalacia, and osteoporosis is mainly mediated by secondary hyperparathyroidism (Figure 2) [1,19]. A low level of serum 25(OH)D results in inadequate intestinal calcium absorption, which, in turn, leads to a transient decrease in serum ionized calcium. This subsequently results in secondary hyperparathyroidism, causing increased bone resorption, which precipitates osteoporosis. Secondary hyperparathyroidism also causes increased urinary phosphate excretion leading to an inadequate calcium-phosphate product, thereby precipitating rickets in children and osteomalacia in adults [1,19].

A low level of serum 25-hydroxyvitamin D causes a significant decrease in the intestinal absorption of calcium and phosphate. This leads to a transient decrease in the serum concentration of ionized calcium and subsequent secondary hyperparathyroidism. Elevated parathyroid hormone induces the differentiation of preosteoclast into mature osteoclast, thereby leading to an increased osteoclast number and activity. This causes increased bone resorption, loss of bone mineral and matrix, and subsequent low bone mass and osteoporosis. Furthermore, parathyroid hormone exhibits a phosphaturic effect, resulting in an increase in urinary phosphate excretion. Urinary phosphate loss along with decreased intestinal phosphate absorption due to vitamin D deficiency contributes to an inadequate calcium−phosphate product, thereby leading to bone mineralization defects and the development of rickets and osteomalacia.

### 3.2. Effects of Vitamin D on the Immune System

**Figure 3:**
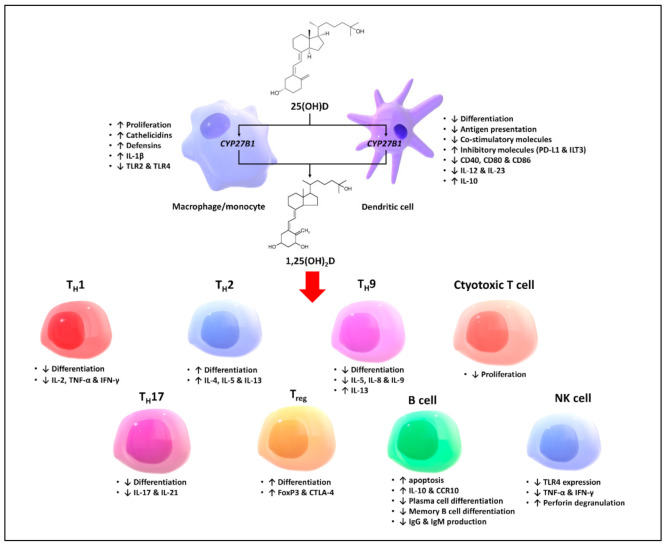
Effects of vitamin D on different cell types of the innate and adaptive immune systems. ↑: Increased; ↓: Decreased; 1,25(OH)_2_D: 1,25-dihydroxyvitamin D; 25(OH)D: 25-hydroxyvitamin D; CCR10: C-C chemokine receptor type 10; CTLA-4: cytotoxic T-lymphocyte-associated protein 4; CYP27B1: cytochrome P450 family 27 subfamily B member 1; FoxP3: Foxhead box P3; IFN-γ: interferon-γ; IgG: immunoglobulin G; IgM: immunoglobulin M; IL-1β: interleukin-1β; IL-2: interleukin-2; IL-4: interleukin-4; IL-5: interleukin-5; IL-8: interleukin-8; IL-9: interleukin-9; IL-10: interleukin-10; IL-13: interleukin-13; IL-17: interleukin-17; IL-21: interleukin-21; ILT3: immunoglobulin-like transcript-3; NK: natural killer; PD-L1: programmed death-ligand 1; TH1: T helper 1; TH2: T helper 2; TH9: T helper 9; TH17: T helper 17; Treg: regulatory T cell; TNF-α: tumor necrosis factor-α; TLR2: toll-like receptor 2; TLR4: toll-like receptor 4.

Vitamin D is known not only for its functions in maintaining calcium and phosphate homeostasis, but also for its immunomodulatory effects on several components of the innate and adaptive immune systems [6,20]. Evidence on the effects of VDR activation on the proliferation, differentiation, and function of each immune cell type is to be reviewed in this section, which is summarized in Figure 3.

Activated macrophages and monocytes, induced by exposure to inflammatory cytokines (e.g., IFN-Ƴ) and toll-like receptor signaling, express CYP27B1, which converts 25(OH)D into 1,25(OH)_2_D, which acts in an autocrine and paracrine fashion to regulate the innate and adaptive immune systems [21]. It has been shown that 1,25(OH)_2_D stimulates macrophage proliferation and the production of the proinflammatory cytokine interleukin-1β, as well as endogenous antimicrobial peptides, cathelicidins, and defensins [22,23]. In the presence of granulomatous inflammation (e.g., TB, sarcoidosis, fungal infections and some lymphomas), an excessive amount of 1,25(OH)_2_D can be produced by the macrophages, causing unregulated increased 1,25(OH)_2_D in the systemic circulation, which results in hypercalcemia and hypercalciuria [24].

1,25(OH)_2_D regulates the functions and differentiation of antigen-presenting cells (APCs) by decreasing the antigen presentation and increasing the expression of inhibitory molecules on the cell surface, causing the APCs to become tolerogenic and more immature [25,26,27]. It does so by decreasing the expression of MHC class II and co-stimulatory molecules, inhibiting the production of IL-12 and IL-23, and stimulating the production of IL-10, a tolerogenic cytokine [25,28,29]. 1,25(OH)_2_D, in addition, is shown to downregulate the expression of toll-like receptors on the monocytes and inhibit the production of proinflammatory cytokines (i.e., IL-2, IL-6, and IL-17) [6,20,30].

1,25(OH)_2_D is known to modulate the adaptive immune system by activating the VDR expressed by the APCs and activated T and B lymphocytes, which, in general, results in a shift of immune status from a proinflammatory to tolerogenic state. 1,25(OH)_2_D inhibits the proliferation of T lymphocytes and regulates cytokine production and differentiation with various effects on different subgroups of T lymphocytes. It promotes a shift from T helper 1 (T_H_1), T helper 9 (T_H_9), and T helper 17 (T_H_17) immune profiles to the T helper 2 (T_H_2) immune profile, and facilitates the differentiation of regulatory T cells (T_reg_) [31,32,33]. Although little is known about the direct effect of 1,25(OH)_2_D on the cytotoxic lymphocytes, it is believed that 1,25(OH)_2_D may suppress the proliferation of cytotoxic lymphocytes based on the observation that the oral administration of high-dose vitamin D_3_ is associated with an increase in the CD4/CD8 ratio [34,35].

Besides its effects on the T lymphocytes, 1,25(OH)_2_D has been shown to have a negative effect on antibody production by the B lymphocytes when in a hyperactive state via multiple mechanisms. It induces the apoptosis of activated B cells and plasma cells, thereby inhibiting plasma cell formation [36,37]. Furthermore, 1,25(OH)_2_D directly promotes the production of anti-inflammatory cytokines, such as interleukin-10 and CCR10, and inhibits the differentiation from mature B cells to memory B cells and plasma cells [38,39,40]. It is therefore believed that, by dampening antibody production, 1,25(OH)_2_D may benefit by reducing the risk and severity for autoantibody-mediated autoimmune disorders such as SLE and type 1 diabetes [41,42].

It is important to note that supplementation of vitamin D or raising serum 25(OH)D is not equivalent to activating the VDR in the immune cells, as circulating 1,25(OH)_2_D is regulated by PTH and FGF-23, and patients with low levels of 25(OH)D may have normal or even a high level of circulating 1,25(OH)_2_D due to secondary hyperparathyroidism [11,43]. However, it is reasonable to postulate that circulating 25(OH)D may be converted into 1,25(OH)_2_D by the enzyme CYP27B1 expressed by the immune cells, where it triggers intracrine and paracrine signaling, as clinical studies have demonstrated changes in immune profiles in response to vitamin D supplementation similar to what is expected from treating the immune cells with 1,25(OH)_2_D in vitro [44,45].

Equally important is the concept of individual responsiveness to vitamin D supplementation, as studies have demonstrated a high inter-individual difference in the genome-wide expression in human peripheral blood mononuclear cells following vitamin D supplementation. In a recent clinical trial by Shirvani et al. [46], approximately 60% of healthy adults with a vitamin D deficiency or insufficiency (25(OH)D < 30 ng/mL or 75 nmol/L) who received 10,000 IUs per day of vitamin D_3_ for 6 months had a robust response in their genome-wide expression compared with the other 40% who had mild to moderate responses, although all subjects increased their serum concentrations of 25(OH)D to the same range of 60–90 ng/mL (150–225 nmol/L). Moreover, they observed that subjects with a robust genomic response to vitamin D supplementation exhibited different patterns of serum metabolomic profiles compared with those with lower degree of responsiveness [47]. This observation is in line with that of the prior study by Carlberg et al. [48], showing robust changes in broad gene expression in about half of the 71 patients with prediabetes who were given 3200 IUs of vitamin D_3_ daily for 5 months. Therefore, it is reasonable to postulate that vitamin D may affect the immune system differently among individuals, which is hypothesized to be due to inter-individual differences in genetic polymorphisms associated with vitamin D metabolism and signaling pathway (e.g., genes encoding VDR, vitamin D binding protein (DBP), and VDR responsive element in the target genes), as well as some undisclosed epigenetic factors [6].

### 3.3. Defining Optimal Serum 25-hydroxyvitamin D

It is still controversial as to what concentration of serum 25(OH)D would provide optimal benefits for bone health and overall health. A serum 25(OH)D concentration of 15 to 20 ng/mL (37.5–50 nmol/L) is considered sufficient for the prevention of rickets and osteomalacia [19]. It is, however, recommended by the Endocrine Society’s Clinical Practice Guideline that serum 25(OH)D concentration should be above 30 ng/mL (75 nmol/L) to maximize the calcemic effects of vitamin D and to minimize the risk of secondary hyperparathyroidism that predisposes for osteoporosis [8]. According to this guideline, vitamin D deficiency and insufficiency are defined as a serum 25(OH)D level of <20 ng/mL (<50 nmol/L) and 20 ≤ 30 ng/mL (50 ≤ 75 nmol/L), respectively [8]. On the other hand, the Institute of Medicine (IOM) concluded that a serum 25(OH)D level of 20 ng/mL is the level necessary for good bone health for practically all individuals [49]. It is also worth acknowledging the historical evidence to postulate vitamin D status in our hunter-gatherer ancestors. It has been reported that indigenous populations in East Africa have serum 25(OH)D in the range of 40–60 ng/mL (100–150 nmol/L) [50]. This range is consistent with that reported in populational-based studies, which is associated with the lowest risk of chronic diseases and all-cause mortality [51]. However, some studies suggest that there may be a U-shaped relationship between 25(OH)D and some adverse outcomes (e.g., mortality, cardiovascular disease, falls, and some cancers) at levels higher than 50 ng/mL (125 nmol/L) [52,53].

### 3.4. Recommended Vitamin D Intake

It is recommended by the Institute of Medicine that children aged ≥1 year old and adults should ingest at least 600 IUs of vitamin D per day to achieve a serum 25(OH)D level of at least 20 ng/mL (50 nmol/L) [49]. The Endocrine Society Clinical Practice Guideline on vitamin D, however, recommends a higher dose of daily vitamin D intake in order to achieve a level of serum 25(OH)D of at least 30 ng/mL (children aged 0–1 year: 400–1000 IUs, upper limit 2000 IUs; children aged 1–18 years: 600–1000 IUs, upper limit 4000 IUs; adults aged >18 years 1500–2000 IUs, upper limit 10,000 IUs) [8]. Some experts also suggest that adults should be on 4000–6000 IUs to maintain a serum 25(OH)D level in the preferred range of 40–60 ng/mL (100–150 nmol/L) [54]. Notably, patients with obesity and intestinal malabsorption require a two to three times higher amount of vitamin D to maintain the same serum 25(OH)D concentrations [8]. In addition, patients who receive chronic glucocorticoid therapy need two to three increased doses of vitamin D intake as glucocorticoids can cause increased catabolism of both 25(OH)D and 1,25(OH)_2_D [8]. Recommendations by the Institute of Medicine and the Endocrine Society Clinical Practice Guideline for the daily intake of vitamin D and for the treatment for vitamin D deficiency are summarized in Table 1.

### 3.5. Vitamin D from Sunlight Exposure and Diets

Humans get vitamin D from sunlight exposure, diet, and supplements. The amount of vitamin D synthesized by the skin is known to be dependent on the intensity and duration of exposure to ultraviolet B radiation at wavelength of 290–315 nm and skin pigmentation. It is estimated that sunlight exposure 1/4 of a minimal erythemal dose (MED) over 1/4 of the body surface area is equivalent to ingestion of oral 1000 IUs of vitamin D_3_ [55]. People living in high-latitude regions are more susceptible to vitamin D deficiency, especially in the wintertime, because of the oblique zenith angle of the sun. It is documented that when living above or below 33° latitude, little or no vitamin D can be produced in the skin during the winter. During the summertime or near the equator, vitamin D can be synthesized effectively only during 10:00 a.m.–15:00 p.m. [1,56]. Given the limited availability of vitamin D from sunlight exposure, many people rely on oral vitamin D intake to achieve vitamin D sufficiency.

It should be noted that only few foods naturally contain vitamin D. These include oily fish (up to 1000 IUs of D_3_/3.5oz), cod liver oil (up to 1000 IUs of D_3_/tsp), sun-dried or ultraviolet-irradiated mushrooms (up to 1000 IUs of D_2_/3.5oz), egg yolk (20 IUs of D_2_ or D_3_), and meat (variable amount in the form of D_3_ and 25(OH)D_3_). Fortified milk, yogurt, and orange juice in the US contains 100 IUs of vitamin D_2_ or D_3_ per serving (8 oz) [1,2]. Taken together, with minimal sunlight exposure, it is difficult to achieve adequate vitamin D intake solely from foods, and, therefore, many individuals may require vitamin D supplementation to prevent vitamin D deficiency and insufficiency.

### 3.6. Screening for Vitamin D Status

The Endocrine Society Clinical Practice Guideline on vitamin D recommends that screening for vitamin D deficiency should be performed in individuals who are at risk for vitamin D deficiency, such as older adults with a history of falls or fractures; patients with chronic illnesses, obesity, and intestinal malabsorption; or individuals taking medications that interfere vitamin D metabolism (e.g., glucocorticoids, antiepileptics, antiretrovirals, and antifungals) [8]. Nonetheless, the US Preventive Services Task Force reported that evidence on the benefits of screening for vitamin D deficiency in the general population is still lacking [57]. It is, however, advisable that patients with chronic inflammatory disorders with or without corticosteroid therapy should be screened and treated for vitamin D deficiency and supplemented with adequate calcium and vitamin D to prevent further bone resorption on top of chronic inflammation-associated bone loss.

## 4. Evidence on Vitamin D for Prevention and Treatment of Rheumatic Diseases

Multitudes of observational studies have demonstrated the association of low level of 25(OH)D with the presence and severity of several rheumatic diseases. However, the benefits of vitamin D supplementation for the prevention and treatment of these diseases tends to be is less accepted, as the association can be explained in part by confounders such as limited physical outdoor activities and sunlight exposure in patients with chronic illnesses. In this section, the relationship between vitamin D and rheumatic diseases and evidence from clinical studies demonstrating the impact of vitamin D supplementation are reviewed, which is summarized in Table 2.

### 4.1. Rheumatoid Arthritis

RA is a chronic inflammatory disease characterized by synovial inflammation causing symmetrical polyarthritis, affecting approximately 4 cases per 10,000 person−years [58]. RA was classically considered a T_H_1-mediated disease [59]. However, recent studies have suggested that increased T_H_17 and T_H_22 activities and dysfunctional T_reg_ also play a role in the pathogenesis of RA [60,61]. Individuals with certain genetic variations of the human leukocyte antigen (HLA) genes are known to be susceptible to RA, while the only well-established environmental risk factor of RA is cigarette smoking [62].

Vitamin D is believed to play a role in modulating the pathogenesis and disease activity of RA, based on the actions of 1,25(OH)_2_D on the adaptive immune response that suppresses the proliferation and activity of T_H_1 and T_H_17 and enhances the T_reg_ activity [63]. Furthermore, genomic studies have shown that certain polymorphisms of the gene encoding VDR and DBP are associated with susceptibility to RA, suggesting that the vitamin D signaling pathway may be involved in the pathogenesis of RA [64,65].

Multiple observational studies have shown the association of vitamin D status or intake with incidence and severity of RA [66]. For example, in a prospective cohort study by Merlino et al., women in the highest tertile of vitamin D intake had a lower risk for RA by 33% compared with those in the lowest tertile [67]. Moreover, a higher amount of ultraviolet B exposure was shown to be associated with a decreased risk of incident RA in the Nurse Health Study cohort of 106,368 women aged 30–55 years old [68]. This finding is in line with the evidence that the risks of some immune-mediated diseases (e.g., type 1 diabetes, multiple sclerosis, and RA) are higher in high-latitude regions where there is a relatively low amount of ultraviolet radiation and a high prevalence of vitamin D deficiency [69,70]. These observations, therefore, support that vitamin D obtained from either oral intake or sunlight exposure could possibly be protective against RA. In the COMOrbidities in Rheumatoid Arthritis (COMORA) study consisting of 1413 patients with RA from 15 countries, the serum level of 25(OH)D was inversely correlated with disease activity, as assessed by the Disease Activity Score-28 (DAS28) after adjusting for potential confounders [71].

Although the observed association between vitamin D status and RA incidence and severity, like in other diseases, could be partly explained by confounders, the results from clinical trials have suggested that giving vitamin D to patients with RA may help mitigate the disease activity. In a meta-analysis of six studies including 438 RA patients, vitamin D supplementation resulted in a significant improvement in the DAS28 (weighted mean difference (WMD) −0.41, 95%CI: −0.59–−0.23), erythrocyte sedimentation rate (WMD −3.40, 95%CI: −6.62–−0.18), and tender joint count (WMD −1.44, 95%CI: −2.74–−0.14), but not in pain visual analog scale [72]. It was also shown in another meta-analysis of two randomized studies that vitamin D supplementation resulted in an insignificant reduction in RA flares, defined by a DAS28 of >3.2 (risk difference −0.10, 95% CI: −0.21–0.00), although a high risk of bias was noted in one of the studies due to the open-label design [73,74]. It should, however, be noted that most of the individual studies included in these meta-analyses did not show a statistically significant benefit of vitamin D supplementation. This could be due to difference in patient characteristics, limited statistical power due to small sample size, and possibly the fact that the doses of vitamin D used in some studies were too low.

In addition, some studies have also shown that giving 1,25(OH)_2_D can also help improve the outcomes of RA. In an open-labeled randomized clinical trial by Gopinath et al. [75], 59 RA patients who received 1,25(OH)_2_D_3_ along with disease-modifying anti-rheumatic drugs (DMARDs) and calcium demonstrated a significantly higher pain relief compared with the 62 patients receiving DMARDs and calcium alone. Another phase II clinical trial by Li et al. [76] gave 22-oxa-1,25(OH)_2_D_3_ or 1,25(OH)_2_D_3_ or placebo to 369 RA patients, and observed a significant reduction in swollen joints and improved Health Assessment Questionnaire Disease Activity Index scores in the groups receiving two active treatments compared with the placebo group.

In summary, there is suggestive observational evidence that increasing vitamin D intake to raise serum 25(OH)D may reduce the risk of developing RA. However, there is no demonstration from a clinical trial that vitamin D supplementation can reduce the risk of incident RA. There is moderate evidence that vitamin D supplement or the oral administration of 1,25(OH)_2_D can mitigate the disease severity of RA. Further large-scale of randomized clinical trials are required before any form of vitamin D or 1,25(OH)_2_D can be recommended as an adjunctive treatment for RA in clinical practice.

### 4.2. Systemic Lupus Erythematosus

SLE is an autoimmune disorder with a broad variety of clinical presentations, including, but not limited to, constitutional symptoms, arthralgias, arthritis, glomerulonephritis, serositis, vascular phenomenon, skin rashes, and hematologic cytopenias [77]. The reported incidence of SLE ranges from 0.3/100,000 person−years in Africa to 23.2/100,000 person−years in North America [78]. The pathogenesis of SLE is not fully understood, but it is hypothesized to involve genetic predisposition and environmental predisposing factors such as cigarette smoking, Ebstein−Barr virus infection, and silica exposure [79]. Patients with SLE have been shown to have dysregulated immune profiles characterized by increased T_H_17 and decreased T_reg_ activities with variable T_H_1 and T_H_2 activities, as well as the development of autoantibodies (e.g., ANA, anti-Sm/RNP, anti-Ro/La, anti-dsDNA, etc.) [80,81,82,83].

A low level of serum 25(OH)D has been shown to be associated with the presence of SLE in several case-control studies [84,85]. In addition, there is evidence from population genetic studies that individuals carrying some genetic polymorphisms of VDR (i.e., BsmI and FokI polymorphic variants) are at an increased risk for SLE [86]. It has also been shown that the expression of VDR in 20 renal biopsy specimens was negatively associated with the Systemic Lupus International Collaborating Clinics (SLICC) renal activity scores and the SLE disease activity index scores (SLEDAI) [87]. Therefore, it can be postulated that the activation of the vitamin D signaling pathway may help mitigate the autoinflammatory process in SLE and lupus nephritis via its immunomodulatory effects on T_H_17, T_reg_, and B cells, and possibly the direct effects on renal tissue.

Nevertheless, in the Nurses’ Health Studies I and II of 186,389 women and 190 incident SLE cases, there was no association between vitamin D intake and risk of incident SLE, indicating that the association between vitamin D status/intake and SLE may not be causal, as no temporal association was demonstrated [88]. On the other hand, evidence on the benefit of vitamin D for alleviating the disease activity of SLE seems to be more recognized, as low levels of 25(OH)D have been shown to be associated with the disease activity of SLE, indicated by the SLE disease activity index scores (SLEDAI), anti-dsDNA positivity, and rate of remission [85]. Moreover, in a meta-analysis of five randomized controlled trials with a total of 490 patients with SLE, vitamin D_3_ supplementation was found to decrease the fatigue severity scale scores in patients with SLE (two trials with 79 patients; standard mean difference −1.179, 95% CI: −1.9–−0.46), although no significant changes in the SLEDAI and positivity of anti-dsDNA were observed [89].

Taken together, it is well-documented that low level of 25(OH)D is associated with SLE occurrence and disease severity. There is evidence from a few clinical trials that vitamin D supplement may improve the disease activity in SLE patients. However, whether improving vitamin D status/intake can reduce the risk of developing SLE needs further investigation.

### 4.3. Spondyloarthropathies

SpA are a family of autoimmune diseases that share certain genetic predisposing factors and clinical characteristics [90]. These include ankylosing spondylitis (AS), reactive arthritis, psoriatic arthritis (PsA), inflammatory bowel disease (IBD), and undifferentiated spondyloarthropathy. These diseases are grouped based on their association with the human leukocyte antigen-B27 (*HLAB27*) gene and the presence of enthesitis as a common clinical feature. Other clinical features include dactylitis, back pain, uveitis, and skin rash [90]. The pathogenesis of SpA is still poorly understood; however, it is hypothesized to involve nonantigen-presenting properties of HLAB27, self- or bacterial-derived antigenic peptides, and gut dysbiosis that trigger autoimmunity [91].

1,25(OH)_2_D is shown to abrogate the osteoclastogenic potential and proinflammatory cytokine secretion capacity of immune cells of patients with psoriasis and PsA [92,93,94]. This could explain the observation that serum 25(OH)D was inversely correlated with presence of PsA [95], as well as plasma C-reactive protein level among patients with PsA [96]. Moreover, some reports have shown marked clinical improvement in patients with psoriasis who received up to 50,000 IUs per day of oral vitamin D_3_ [97,98,99]. However, the benefit of lower doses of up to 4200 IUs per day or equivalent of vitamin D supplements for the treatment of psoriasis is still unverified based on the results of a few clinical trials [100].

Besides PsA, low levels of serum 25(OH)D have been observed in patients with AS [101] and IBD [102] compared with healthy individuals. Vitamin D insufficiency (25(OH)D < 30 ng/mL) and deficiency (25(OH)D < 20 ng/mL) were shown to predict all-cause mortality among patients with AS in a populational based study of 919 Israeli patients [103]. It is also worth noting that vitamin D supplementation has been shown in randomized clinical trials to improve outcomes in patients with IBD and to alter the composition of gut microbiota towards genera associated lower inflammatory burden [104,105]. Thus, vitamin D is believed to play a role in modulating the disease severity of SpA through its effects not only on the immune cells, but also on the gut microbiota, which is thought to play a role in the pathogenesis of SpA. Nevertheless, clinical trials investigating the impact of vitamin D supplementation in patients with SpA are still lacking.

Interestingly, DBP (*GC*) gene polymorphisms were shown to be associated with the development of peripheral arthritis and uveitis in 223 Korean patients with AS [106]. There is also a case report of a patient who had concomitant homozygous deletion of the DBP and deliberating AS with relatively mild disruption of bone metabolism [107]. Provided the evidence that DBP has pleiotropic functions in sequestration of actin and a variety of less-defined roles in modulating immune responses [108], DBP may be a more significant mediator of the disease, and that the observed association between AS and vitamin D is, in fact, due to the variation in circulating DBP that is correlated with the measured serum 25(OH)D.

### 4.4. Gout and Hyperuricemia

Gout is a systemic disease characterized by the deposition of monosodium urate crystals in the tissues. This condition requires increased serum uric acid above a specific threshold to form uric acid crystals [109]. Although hyperuricemia is the major predisposing factor in gout, only about 5% of individuals with hyperuricemia above 9 mg/dL develop gout [109]. In a meta-analysis of seven cross-sectional studies, individuals with vitamin D deficiency (25(OH)D < 20 ng/mL) and insufficiency (25(OH)D 20– < 30 ng/mL) have been shown to have increased serum uric acid in a dose-dependent manner compared with vitamin D-sufficient individuals (pooled mean differences 0.45 and 0.33 mg/dL, respectively) [110]. The association is thought to due, not only to the fact that both vitamin D deficiency/insufficiency and hyperuricemia share common comorbidities such as obesity and metabolic syndrome, but also to a direct causal association between the two conditions. This is supported by the study of 71 patients with prediabetes who were randomized to receive weekly doses of 20,000 IUs of vitamin D_2_, 15,000 IUs of vitamin D_3_ or no vitamin D, that vitamin D supplementation was associated with a reduction in mean serum uric acid level by 0.6 mg/dL in those with baseline uric acid level of >6 mg/dL [111]. It has been suggested that the mild uric-lowering effect of vitamin D is mediated by the suppression of PTH, which is known to downregulate the ATP-binding cassette transporter G2 (ABCG2) in the kidneys, leading to a reduction in the renal clearance of uric acid [112,113]. Furthermore, studies have shown that patients with primary hyperparathyroidism had increased serum uric acid [114] and that those who underwent parathyroidectomy had decreased serum uric acid levels postoperatively [115,116], indicating a significant effect of PTH on serum uric acid. However, there has been no demonstration of whether vitamin D supplementation can reduce urinary uric acid excretion. Despite the causal link between vitamin D and uric acid, vitamin D status was not found to be associated with gout in the populational-based data from the US National Health and Nutrition Examination Survey (NHANES) [117].

Based on the current evidence, correcting vitamin D deficiency/insufficiency has a mild uric-lowering effect (~0.3–0.6 mg/dL), which is thought to be mediated by the suppression of PTH. However, no direct association between vitamin D and gout has been demonstrated.

### 4.5. Osteoarthritis

OA is the most common degenerative joint disease and a major cause of pain and disability affecting more than 25% of adults aged 65 years or more [118]. The interplaying mechanisms of OA include articular cartilage degradation, osteophyte formation, subchondral sclerosis, and synovial hyperplasia [119]. Risk factors of OA include joint injury, aging, obesity, and genetics [120].

Given that low level of serum 25(OH)D has been shown in some studies to be associated with the presence and severity of knee osteoarthritis in both younger and older individuals [121,122], it is estimated that vitamin D may affect the development and progression of OA due to its impacts on not only bone quality, but also pain reduction, due to reduced inflammation and improved skeletal muscle function of the lower extremities. This can be supported by the evidence from clinical trials showing the benefit of vitamin D for the improvement of muscle strength, body sway, and physical performance, which is thought to be due to the genomic and non-genomic actions of 1,25(OH)_2_D on energy metabolism and the function of the skeletal muscle [123,124].

A randomized controlled trial by Sanghi et al. [125] in 107 patients with knee OA demonstrated that giving 60,000 IUs daily followed by 60,000 IUs monthly of oral vitamin D_3_ for 12 months resulted in a significant decrease in visual analog scale pain (between−group mean difference (MD) −0.39, 95%CI: −0.71–−0.08) and total Western Ontario and McMaster Universities Arthritis Index (WOMAC) (MD −3.53, 95%CI: −4.39–−2.71) compared with placebo. In a study by Jin et al. [126], 413 patients with knee OA were randomized to receive either 50,000 IUs of vitamin D_3_ per month or placebo for 2 years. Compared with the placebo group, the group receiving vitamin D_3_ was found to have significant improvements in WOMAC function (MD −72.9, 95%CI: −126.4–−19.4) and total WOMAC (MD −91.4, 95%CI: −165.1–−17.7). However, no significant difference between the groups in the tibial cartilage volume or WOMAC pain was observed in this study. In other clinical trials, no significant effect of vitamin D on the outcomes of knee OA was observed [127,128]. In sum, vitamin D supplement may have a modest effect on improving pain and function in patient with knee OA; however, it does not reverse the disease process.

### 4.6. Other Rheumatic Diseases

Multiple observational studies have revealed an association between a low level of serum 25(OH)D and the presence and/or severity of several rheumatic diseases, including systemic sclerosis, inflammatory myopathies, and vasculitis [129,130,131,132,133]. However, evidence from clinical trials demonstrating the impact of any form of vitamin D on most of these diseases is still lacking. It is therefore still unclear if the association between vitamin D and these conditions is causal or more likely explained by confounders and reverse causation, such as limited physical activity or corticosteroid use. In a pilot clinical trial of 20 patients with localized scleroderma, 9-month oral calcitriol therapy (0.75 µg/day for 6 months followed by 1.25 µg/day for 3 months) was not more effective than the placebo in the improvement of the skin score [134]. It should be noted that patients presenting with chronic widespread pain due to osteomalacia caused by vitamin D deficiency can often fulfill the clinical criteria for the diagnosis fibromyalgia [135]. This may partially explain the observed pain reduction benefit of vitamin D in some, but not all clinical trials [136], as some of the patients who had osteomalacia mimicking fibromyalgia might have been treated.

**Table 2:** Main characteristics of studies demonstrating the impact of vitamin D supplementation on clinical outcomes in patients with rheumatic disease.

Disease	First Author and Year of Publication	Study Design/Intervention/Studied Participants	Main Results
RA	Guan et al., 2020 [72]	A meta-analysis of 6 randomized controlled trial investigating the efficacy of 8000–50,000 IUs per week of oral vitamin D or equivalent or oral 1,25(OH)_2_D on disease activity in patients with RA (total *n* = 438).	Vitamin D supplementation resulted in a significant improvement in the DAS28 (WMD −0.41, 95%CI: −0.59–−0.23), ESR (WMD −3.40, 95%CI: −6.62–−0.18) and tender joint count (WMD −1.44, 95%CI: −2.74–−0.14) but not in pain VAS.
	Nguyen et al., 2020 [73]	A subgroup meta-analysis of 2 randomized controlled trial investigating the efficacy of oral 50,000 IUs/week of vitamin D_3_ or 0.5 mcg/day of alfacalcidol [1α-hydroxyvitamin D_3_] on the risk of flare in RA patients in remission (total *n* = 252).	Vitamin D supplementation resulted in an insignificant reduction in RA flares defined by the DAS28 of >3.2 (risk difference −0.10, 95% CI: −0.21–0.00).
SLE	Zheng et al., 2019 [89]	A meta-analysis of 5 randomized controlled trials investigating the efficacy of 200–50,000 IUs/week of vitamin D_3_ or equivalent on disease activity in patients with SLE (total *n* = 490).	Vitamin D_3_ supplementation resulted in a decrease in the fatigue severity scale scores in patients with SLE in a meta-analysis of 2 trials (*n* = 79; SMD −1.179, 95% CI: −1.90–−0.46). However, no significant changes in the SLEDAI and positivity of anti-dsDNA was observed.
Hyperuricemia	Nimitphong et al., 2021 [111]	A randomized controlled trial giving either 20,000 IUs/week of vitamin D_2_, 15,000 IUs/week of of vitamin D_3_ or placebo to patients with prediabetes (*n* = 71).	Among patients with baseline serum uric >6 mg/dL (*n* = 36), associated with a reduction in mean serum uric acid level by 0.6 mg/dL.
OA	Sanghi et al., 2013 [125]	A randomized controlled trial giving either 60,000 IUs of vitamin D_3_/day for 10 days followed by 60,000 IUs/month for 12 months or placebo to patients with knee OA with serum 25(OH)D <20 ng/mL (*n* = 107).	Vitamin D_3_ supplementation results in a significant decrease in visual analog scale pain (between-group MD −0.39, 95%CI: −0.71–−0.08) and total WOMAC (MD −3.53, 95%CI: −4.39–−2.71).
	Jin et al., 2016 [126]	A randomized controlled trial giving either 50,000 IUs/week of vitamin D_3_ per day for 2 years or placebo to patients with knee OA with serum 25(OH)D <24 ng/mL (*n* = 413).	Vitamin D_3_ supplementation resulted in significant improvements in WOMAC function (MD −72.9, 95%CI: −126.4–−19.4) and total WOMAC (MD −91.4, 95%CI: −165.1–−17.7). However, no significant difference between groups in tibial cartilage volume or WOMAC pain was observed.
Chronic widespread pain/fibromyalgia	Yong et al., 2017 [136]	A meta-analysis of 4 randomized controlled trials investigating the efficacy of 25,000–75,000 IUs/week of oral or intramuscular vitamin D on pain VAS in patients with chronic widespread pain or fibromyalgia (total *n* = 287)	Vitamin D supplementation resulted in a significantly lower pain VAS (pooled MD 0.46, 95%CI 0.09–0.89).

1,25(OH)_2_D: 1,25-dihydroxyvitamin D; 25(OH)D: 25-hydroxyvitamin D; 95%CI: 95% confidence interval; DAS28: Disease Activity Score-28; ESR: erythrocyte sedimentation rate; MD: mean difference; OA: osteoarthritis; RA: rheumatoid arthritis; SMD: standardized mean difference; SLE: Systemic Lupus Erythematosus; SLEDAI: Systemic Lupus Erythematosus Disease Activity Index; VAS: visual analogue scale; WMD: weighted mean difference; WOMAC: The Western Ontario and McMaster Universities Arthritis Index.

## 5. Conclusions

Vitamin D plays an essential role in not only maintaining healthy mineralized skeleton, but also modulating the innate and adaptive immune systems in a way that is thought to benefit as an adjunctive treatment for many immune-mediated diseases. A low level of 25(OH)D is associated with the presence and severity of most, if not all, rheumatic diseases, such as RA, SLE, SpA, and OA. However, the benefits of vitamin D supplement for the treatment and prevention of these diseases are relatively unclarified, as the results from existing clinical trials are markedly inconsistent. Many of them are small in sample size and likely underpowered; however, when those results were pooled in meta-analyses, there were conceivable signals of the benefits of vitamin D for the improvement of disease activity, particularly for RA and SLE. It is also worth noting that, based on recent genomic studies on vitamin D, there might be inter-individual difference in responsiveness to vitamin D supplementation that need further investigations, suggesting that some individuals might be able to benefit from vitamin D more or less than others. Regardless of the evidence on the disease-specific benefits of vitamin D, it is advisable that patients with rheumatic disease with or without corticosteroid therapy should have sensible sunlight exposure and adequate vitamin D intake to maintain serum 25(OH)D level at least 30 ng/mL (75 nmol/L) in order to prevent osteomalacia, secondary osteoporosis, and fracture, and possibly 40–60 ng/mL (100–150 nmol/L) to achieve the maximal benefit from vitamin D for immune health and overall health.

## Figures and Tables

**Table 1:** Recommendations for daily intake of vitamin D and treatment for vitamin D deficiency.

Population	The Institute of Medicine’s Recommendations [49]			The Endocrine Society Clinical Practice Guideline on Vitamin D’s Recommendations [8]		
	Adequate Intake	Estimated Average Allowance Per Day	Recommended Daily Allowance	Daily Requirement	Upper Limit Per Day	Treatment for Vitamin D Deficiency
0–1 year	400 IUs	-	-	400–1000 IUs	2000 IUs	2000 IU/d or 50,000 IU/wk of vitamin D_2_ or D_3_ for at least 6 weeks to achieve serum 25(OH)D >30 ng/mL (75 nmol/L) maintenance therapy of 400–1000 IU/d
1–18 years	-	400 IUs	600 IUs	600–1000 IUs	4000 IUs	2000 IU/d or 50,000 IU/wk of vitamin D_2_ or D_3_ for at least 6 weeks to achieve serum 25(OH)D >30 ng/mL (75 nmol/L) maintenance therapy of 600–1000 IU/d
18–70 years	-	400 IUs	600 IUs	600–1000 IUs	10,000 IUs	6000 IU/d or 50,000 IU/wk of vitaminD_2_ or D_3_ for 8 weeks to achieve serum 25(OH)D >30 ng/mL (75 nmol/L) maintenance therapy of 1500–2000 IU/d
>70 years	-	400 IUs	800 IUs	1500–2000 IUs	10,000 IUs	
Adult patients with abnormal kinetics of vitamin D *	-	-	-	6000–10,000 IUs	10,000 IUs	Dosage should be increased by 2–3 times

* Patients with abnormal kinetics of vitamin D include those with obesity or intestinal malabsorption, or those taking medications that increase the catabolism of vitamin D, including corticosteroids, antiseizures, antiretrovirals, etc.

## Data Availability

Not applicable.
